# Supplementary material for: Widely tunable and narrow-linewidth violet lasers enabled by UV-transparent materials
Source: Nat Commun. 2025 Nov 21;16:10294. doi: 10.1038/s41467-025-65211-2 (PMC12638939; doi:10.1038/s41467-025-65211-2)
Supplement: Supplementary file 1 — Supplementary Information [file 41467_2025_65211_MOESM1_ESM.pdf]

Widely tunable and narrow-linewidth violet lasers enabled by UV transparent materials

C.A.A. Franken[1,*], W.A.P.M. Hendriks[2,3], L.V. Winkler[1,4], A.R. do Nascimento Jr.[5], A. van Rees[1], M. Dijkstra[2], S. Mardani[2], D. Kienzler[6,7], R. Dekker[8], J. van Kerkhof[5], P.J.M. van der Slot[1,9], S.M. García-Blanco[2,3], and K.-J. Boller[1]

[1] *Laser Physics and Nonlinear Optics, Department of Science and Technology, MESA+ Institute of Nanotechnology, University of Twente, Enschede, The Netherlands*
[2] *Integrated Optical Systems, Department of Science and Technology, MESA+ Institute of Nanotechnology, University of Twente, Enschede, The Netherlands*
[3] *Aluvia Photonics B.V., Enschede, The Netherlands*
[4] *TOPTICA Photonics SE, Gräfelfing, Germany*
[5] *PHIX B.V., Enschede, The Netherlands*
[6] *Department of Physics, ETH Zurich, Zurich, Switzerland*
[7] *Quantum Center, ETH Zurich, Zurich, Switzerland*
[8] *LioniX International B.V., Enschede, The Netherlands*
[9] *Nonlinear Nanophotonics, Department of Science and Technology, MESA+ Institute of Nanotechnology, University of Twente, Enschede, The Netherlands*
[*] *Corresponding author, e-mail: c.a.a.franken@utwente.nl*

Supplementary Information

Supplementary Note 1. Feedback chip design

Choosing a proper waveguide cross-section is of central importance to design low-loss and fabrication tolerant waveguide components. This applies particularly to curved waveguides in small ring resonators for spectral filtering with wide free spectral range, where the cross-section and ring radius determines the bending loss and thus the filter intrinsic Q-factor. Also the design of directional couplers depends on the cross-section, where the coupling strength is influenced by the coupler length and takes in the fabrication tolerance of the gap between the waveguide cores. The cross-section to be selected also needs to restrict propagation to a single transverse mode with a polarization matching that of the amplifier (fundamental TE mode). Efficient coupling to the diode amplifier and output fibers is realized through mode matching via tapering the width of the waveguide, using the modefield diameter as specified by the manufacturers.

The gain bandwidth and its maximum value determines the Vernier filter's free spectral range and the overall allowed feedback loss, while the amplifier's mode profile determines what minimum loss can be achieved in edge coupling and hybrid integration of the chips. By including multiple variants of the same circuit with different values of the bus-to-ring coupler length on the wafer, the optimal circuit can be chosen for packaging after initial characterization of the various test structures. Here, optimal refers to the most promising circuit in terms of sufficiently low laser threshold, widest tuning, and narrowest laser linewidth. With experimentally obtained values for the propagation loss α and bus-to-ring coupling strength for both rings the transmission function of the Vernier filter can be calculated, which is plotted in Fig. 2a (main text). It can be seen that the two individual resonators with a free spectral range of 97 and 99 pm, respectively, open a Vernier free spectral range that is much wider, about 4.9 nm. This ensures that the filter transmits only a single wavelength with low loss within the amplifier emission spectrum, qualitatively represented by the dashed outline of the measured amplified spontaneous emission (ASE) spectrum in Fig.

2a (main text). For light passing resonantly through the Vernier filter, we calculate an optical roundtrip length of the laser cavity of 24 mm. This corresponds to an estimated cavity FSR of at least 12.5 GHz, where the effective roundtrip length, and the cavity FSR, decreases further away from the main Vernier resonance.

Using the on-chip metal heaters, elements in the circuit such as the phase section, outcoupler and ring resonators can be tuned. Tuning the phase section allows for spectrally aligning a laser cavity mode with a resonance of the Vernier filter. Tuning of ring resonators (R_1 and R_2) selects a particular feedback wavelength. The main purpose of tunable outcoupling is to enable maximum output power at each drive current of the laser diode and heaters of the Vernier filter. Tunable outcoupling is achieved by heating one of the arms of the tunable outcoupler using the on-chip heaters. To reduce ASE noise in the output, the output is taken from the filtered light from the Vernier loop mirror that is directed back to the laser amplifier. The phase section behind the outcoupler may be used to prevent injection locking of the laser frequency via unwanted reflections such as from chip and fiber facets. For the measurement reported here, no unwanted reflections have been observed and this phase section remained unused.

Supplementary Note 2. Fabrication

The Al_2O_3 waveguide fabrication, carried out in the Nanolab cleanroom of the MESA+ Institute (University of Twente) within the Integrated Optical Systems group, starts by depositing a 110-nm Al_2O_3 layer using an optimized RF reactive sputter deposition process [1], onto an 8-μm thick thermally oxidized 10-cm diameter silicon wafer. A chemical mechanical polishing step is used to reduce the surface roughness of the deposited Al_2O_3 layer, reducing the layer thickness to the targeted 100 nm. The residual surface roughness is below the resolution of our AFM, we estimate a maximum roughness of 0.2 nm. Next, the substrate is coated with negative e-beam resist (AR-N 7520). Using a Raith EBPG5150 e-beam lithography system the waveguide layer is written in the resist. After the e-beam exposure, the pattern is developed using AR-300-47 developer. The resulting patterns are etched into the Al_2O_3 using an Oxford PlasmaPro 100 Cobra (reactive ion etching). Afterwards the resist is stripped by oxygen plasma using a TePla 300 plasma etcher. The resulting waveguides are fully buried by an 8-μm thick SiO_2 cladding. To implement thermo-optic tuning at various locations on the feedback chips, resistive heaters are fabricated, by deposition and structuring of a 10/10 nm Cr/Pt layer topped with a 300 nm Au layer. All three layers are patterned using a lift-off process. To create the heaters, the gold layer is etched away leaving only the two thin and high electrically resistive Cr and Pt layers. We note that the deposition of electrodes for an integrated ion trap on the same chip may be done in parallel with fabrication of the heater electrodes. For the Al_2O_3 thin film the material index was measured using an ellipsometer (Woollam M-2000UI) in the wavelength range from 600 - 1600 nm. Cauchy's equation, $n(\lambda) = A + B/\lambda^2$ with λ in μm, was fitted to the ellipsometer data with $A = 1.6848 \pm 0.009$ and $B = 0.0119 \pm 0.002\ \mu m^2$. The function was extrapolated to obtain the material index in the spectral range near the UV.

Supplementary Note 3. Characterization of Al_2O_3 feedback chips

Several of the fabricated chips contain test structures to characterize individual building blocks of the waveguide feedback circuit. First, to characterize the straight propagation loss, several spirals and straight waveguides were investigated. These structures use a minimum bend radius of 150 μm, which ensures negligible bend radiation loss while maintaining a small footprint circuit. Using a 405 nm Fabry-Pérot diode laser (QPhotonics, QFLD-405-20S) light was fiber coupled (PM-S405-XP) into the chip, passing through a spiral or path (Fig. 1c main text). At the output side of the chip the transmitted power was measured (Thorlabs, fiber-coupled S150C photodiode). The transmittance (P_{out}/P_{in}) as a function of the on-chip propagation length is shown in Sup-

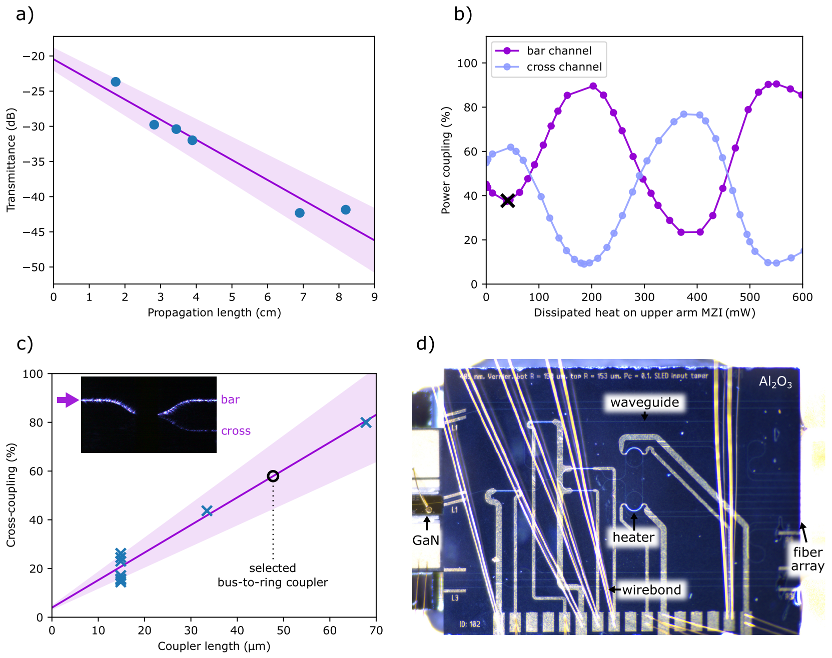

Supplementary Figure 1: **a)** Transmittance of 405 nm light through various paths and spirals of different propagation length. The linear slope indicates a propagation loss of 2.8 ± 0.3 dB/cm and from the y-axis crossing a 10.2 ± 0.8 dB fiber-to-chip coupling loss is obtained from the unpolished Al_2O_3 facets. The shaded area indicates the standard deviation error of the fit. **b)** Power coupling of a test tunable coupler, which consists of Mach-Zehnder Interferometer, as a function of dissipated heat on the upper arm. A 2π phase shift is obtained with about 350 mW of heat on the Cr/Pt heater with a room-temperature resistance of 356 Ohm. The cross marker denotes the setting of the MZI outcoupler in the circuit during the linewidth measurements, about 38% of the light is coupled out of the laser. **c)** Transmission measurement using a set of directional couplers of different lengths were preformed. Feedback circuits with 48 μm long bus-to-ring couplers were selected for integration with a GaN amplifier. **d)** Top-down picture of the packaged, hybrid laser, showing the waveguides, heaters, wire bonds and location of the GaN amplifier and location of the fiber array in detail.

plementary Fig. 1a. Fitting a linear function through the measured points yields a propagation loss of 2.8 ± 0.3 dB/cm and a fiber-to-chip coupling loss of 10.2 ± 0.8 dB/facet. The shaded area in Supplementary Fig. 1a shows the standard deviation error of the linear fit. The error in the facet loss is significant, we contribute this to the large variation in surface roughness at the facet. As these chips are measured with unpolished facets and at a small wavelength, the Rayleigh scattering loss at this interface can be quite large. The error bars for the individual measurements correspond to the error of the powermeter ($\sim$ 0.4 dB) and mode mismatch due to the limited stage accuracy ($\sim$ 0.15 dB). To estimate the propagation loss of the tapers themselves, test structures were investigated where light propagates sequentially through a number of lateral tapers lined up as a waveguide and the measurements indicate that the loss of the tapers is below the measurement accuracy of the set-up.

To determine the phase tuning of light using the fabricated heaters, all of which have the same length and width, another test structure with a Mach-Zehnder interferometer and a thermal heater on each arm is measured. It shows that about 350 mW of electrical power is needed for a phase shift of 2π in the guided light, or phase shifting efficiency of 175 mW/π (see Supplementary Fig. 1b). Furthermore, this test structure showed that our tunable outcoupler can vary the amount of outcoupling between 10 and 90%.

Scanning electron microscope (SEM) images obtained using a focused ion-beam (FIB) set-up allowed us to quantify the waveguide cross-section, the size of the gaps in directional couplers

and tapers in various parts of the photonic chip. These cross-sectional images also verify that the waveguide sidewall is at the designed right angle with the base (90 ± 3 °).

To finalize the characterization of the chips an important parameter for the Vernier filters was measured, namely the coupling strength of the bus-to-ring couplers. Several test structures were measured containing directional couplers with a different length of the waveguides in the coupling region, which should result in different cross-coupling. By measuring the power of light in the bar- and cross-channel, the cross-coupling coupling is obtained (Supplementary Fig. 1c). As expected, the cross-coupling increases with increasing coupling length. For the lasers in this work feedback circuits with a coupling length for the bus-to-ring coupler of $L_{DC} = 48$ μm was chosen. Even though a direct measurement of this coupler was not possible due to damaged facets, the linear trend in Supplementary Fig. 1c indicates a cross-coupling for the $L_{DC} = 48$ μm coupler of 0.58 ± 0.14.

Supplementary Note 4. Hybrid integration and packaging

The hybrid integration and packaging process was carried out in conjunction with PHIX B.V. and with support from Lionix International B.V. First the individual Al_2O_3 chips were diced from the wafer and polished. The fiber output side of the feedback chip (Supplementary Fig. 1d) was polished at an 8° vertical angle to match the angle of the fiber array, minimizing back reflections into the lasing mode from this interface. Here, the waveguide width is tapered down to 120 nm to maximize the mode matching with the large and circular 3.3-μm diameter mode of the PM-S405-XP fibers, showing a theoretical coupling efficiency of 62%, limited by the large difference in mode field diameters of the on-chip and fiber mode. Here, a fiber array with five of these fibers was used.

The gain in the laser is provided by an InGaN/GaN double pass amplifier (superluminescent diode, SLED, from Exalos AG) at 405 nm center wavelength. To work as a double pass amplifier, the diode is highly-reflective coated (reflectivity vs. air >95%) on its back facet and anti-reflective coated (reflectivity vs. air <0.1%) on the facet facing the Al_2O_3 chip. The ASE emission bandwidth of the amplifier was used to set the radii of the Vernier resonators to (radius $R_1 = 150$ μm and radius $R_2 = 153$ μm), to obtain a Vernier spectral range of 4.9 nm selecting a single wavelength in the 3-dB ASE bandwidth (see Fig. 2a main text). Experimental data for the amplifiers are available from the manufacturer. When operated without feedback and driven with a current of 78 mA the output is 10 mW of ASE with a 3-dB emission bandwidth of approximately 3.4 nm. The manufacturer specified mode field diameter is 1.87 by 0.6 μm (width and height). Using 2D mode overlap calculations (Lumerical), a 2.45 μm wide taper on the Al_2O_3 is designed for a theoretical power coupling of about 91% between the GaN amplifier and Al_2O_3 mode. We calculate that for this taper a 100-nm transverse misalignment in the x- and y-direction results in a 1% and 7% reduced coupling efficiency, respectively. Such a 100-nm misalignment is within the specified accuracy of the mechanical stages as used during the chip integration process. For the active temperature control a 10 kΩ NTC thermistor was added to the amplifier submount.

After preparing both chips, the amplifier is turned on at a low current to generate ASE, aligned and edge-coupled to the Al_2O_3 feedback chip (Supplementary Fig. 1d). We optimize the alignment of the chips by maximizing the amount of ASE transmitted through the feedback chip (collecting light with a fiber) without reaching laser threshold (low amplifier current), before the chips are hybrid integrated by bonding using a UV curable epoxy. The epoxy is applied such that the near UV optical mode remains free from epoxy. On the output side of the feedback chip the fiber array is aligned for maximum coupling and bonded using an epoxy, again ensuring an adhesive free optical path between the feedback chip and fibers. Afterwards the full assembly is placed in a standard 14-pin butterfly package. Inside the butterfly package a Peltier element, to be used with the thermistor for the thermal control of the laser, is already mounted and soldered to two pins on the package. The electrical connections for the cathode and anode of the amplifier and heaters on the Al_2O_3 feedback chip are wire bonded to the pins of the butterfly package.

The final step is hermetic sealing of the butterfly package. For each package a getter material

is attached to the inside of the lid before hermetically sealing the package. The getter material absorbs any residual gas (from outgassing of the epoxy, like volatile organic compounds and moisture after sealing). The laser in the pictures (Fig. 1d and 1e main text, Supplementary Fig. 1d) is a package with a glass lid in a nitrogen environment. All of the measurements shown in this work are carried out with a seamwelded metal lid, argon atmosphere laser.

Supplementary Note 5. Experimental details

Equipment

The waveguide heaters are driven with a high-precision, low-noise, multichannel power supply (Chilas B.V., Tunable Laser Controller, TLC). The TLC also contains a PID control loop which controls the temperature of the laser using the thermistor and Peltier element in the butterfly package. The TLC is equipped with a USB interface to receive serial commands from a PC. Together with in-house software, photodiodes and other lab equipment, optimization and control of the laser can be fully automated. Depending on availability several current sources were used: Toptica DLC Pro, Thorlabs LDC205B and ILX Lightwave LDX-3620a. The latter was used for all the linewidth measurements.

Output power

Output powers are measured by connecting the output fiber to a calibrated photodiode (Thorlabs, S150C photodiode). The calibration accuracy of the photodiode is 5% in this spectral range. A 405-nm 90/10 custom fiber splitter (Thorlabs, S405-XP fiber) is used for simultaneous monitoring of spectral properties and output power. To find the conversion for the fiber-coupled output power to the on-chip power, we first note the measured fiber-to-chip coupling loss, which is 10.2 ± 0.8 dB (Supplementary Fig. 1a). This value is measured with an unpolished chip facet in a fiber-chip-fiber set-up, whereas the facets for the final, integrated laser are finely polished which can reduce the fiber-to-chip coupling loss by an estimated 3.5 ± 1.1 dB [2]. Finally, the 5% (∼ 0.2 dB) measurement error for the photodiode can be included in this conversion error. These estimates bring the total loss from chip to fiber to a value of T_{c2f} = -6.7 ± 2.1 dB. Therefore, we find an on-chip power of about 3.5 mW (± 50%).

The mode coupling efficiency from the amplifier to the Al_2O_3 chip depends on mode mismatch, alignment error, and remaining scattering. If the same alignment accuracy is assumed for the amplifier-to-chip interface as for the chip-to-fiber interface, an estimate can be made for the amplifier-to-chip coupling efficiency. The chip-to-fiber transmission is measured to be T_{c2f} = -6.7 ± 2.1 dB = 21 %, where the theoretical coupling efficiency from mode simulations was predicted at 62 %. The theoretical coupling efficiency for the amplifier-to-chip interface is higher than the chip-to-fiber interface at 91 %. Therefore we can approximate an amplifier-to-chip coupling efficiency of $T_c = 0.21 \cdot 0.91/0.62$ = 31 % or -5.0 dB, with a similar error as the chip-to-fiber coupling of ± 2.1 dB.

Optical spectra

The measurements for the spectra, wavelength stability and spectral linewidth are all recorded at the maximum current of 90 mA. Laser output spectra are recorded with an optical spectrum analyzer (Ando AQ6315A). This spectrum analyzer has a resolution of approximately 50 pm or 90 GHz resolution at 405 nm, which should allow us to identify single ring resonances (FSR ∼98 pm). To measure the optical spectra the 90/10 fiber splitter is used to simultaneously monitor the output power of the laser with a photodiode, which enables to use our optimization software for controlling the laser. For comparison, the measured power in all spectra is scaled to the fiber-coupled output power of the laser as calculated from the measurement by the photodiode.

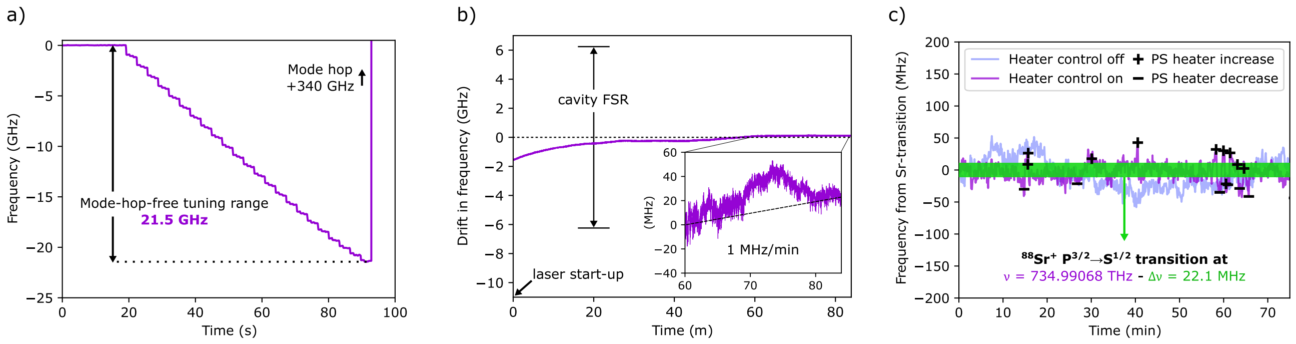

Supplementary Figure 2: **a)** Measurement of the maximum mode-hop-free tuning range. Here the phase section is tuned serially with the both ring resonators. The step size of the ring heaters is 1 mW, and each heater is adjusted sequentially in time steps of 1 s. **b)** High-resolution wavelength meter recording the laser wavelength over time revealing a high passive stability. Recording begins right after start-up, when the laser is still far from thermal equilibrium. After an initial drift over 1.5 GHz, the frequency becomes constant with small residual fluctuations in the order of a few tens of MHz. Here, the average drift comprises to a low 1 MHz/min, without any active frequency stabilization. **c)** Frequency stability of the laser when set to the $^{88}\mathrm{Sr}^{+}$ $\mathrm{P}^{3/2} \rightarrow \mathrm{S}^{1/2}$ transition frequency (averaged per 5 s). The blue trace denotes the passive stability without any feedback (laser heater control turned off, fixed heater currents at the micro-rings and phase section). The purple trace shows the stability with the laser heater control turned on and using incidental, manual adjustments of the phase section heater (about ten-times within an hour) with the smallest possible heater step of 0.15 mW. The + and − markers denote when the phase section heater was adjusted for higher or lower frequency, respectively. The green horizontal band depicts the natural linewidth of the Sr-transition.

Mode-hop-free tuning

Coarse tuning as shown as shown in the previous subsection and in Fig. 2d in the main text, is achieved by tuning *one* of the ring resonator thermo-optic phase shifters. This allows for a fast and full exploration of the tuning bandwidth of the laser, however this coarse tuning is achieved through mode hops of the laser. Alternatively, precise mode-hop-free tuning can be achieved by using serially tuning several thermo-optic phase shifters. Tuning the phase shifters on the phase section and both the micro-ring resonators (Fig. 1b main text) a wide mode-hop-free tuning range is achieved. Here, the ratio of tuning is 5.2:1 for the phase section with respect to both micro-ring resonators. This ratio was found empirically and yielded a maximum tuning range of 21.5 GHz (Supplementary Fig. 2a). The smallest step each heaters could be tuned is about 0.3 mW, limited by the digital-to-analog converter in the heater controller. Besides tuning the phase section together with the ring resonators, fine mode-hop-free tuning can be achieved by only tuning the phase section (Fig. 2e main text). The laser frequency during these measurements was recorded with a calibrated, high resolution wavelength meter (High Finesse WS6-200, absolute accuracy 200 MHz, resolution 4 MHz).

Long-term frequency stability

A mode hop changes the laser frequency by at least one free spectral range of the laser cavity, here about 12.5 GHz, and immunity is present if the longer-term frequency drift remains within that range. A recording, just after laser start-up using a high-resolution wavelength meter shows that the laser was oscillating mode-hop free for at least 84 minutes (Supplementary Fig. 2b), as the frequency drift is well below the cavity FSR. Within the last 24 minutes, a maximum drift of about 40 MHz is measured and the average drift comprises a low 1 MHz/min, which we address to reaching thermal equilibrium with the lab environment. We attribute the high stability to integration of the chips and hermetic sealing and packaging, which includes thermal stabilization. Long-term measurements of passive laser frequency stability rely on a high-resolution wavelength

meter (HighFinesse WS-U 1645, sample rate of ∼100 Hz, resolution of 0.5 MHz).

Precise frequency control

The laser can also be set to a precise wavelength of choice, within the coarse tuning range of the laser, such as the $^{88}Sr^{+}$ $P^{3/2} \rightarrow S^{1/2}$ transition frequency (at ν = 734.99068 THz or λ = 407.88650 nm). The procedure to reach a wavelength of choice is as follows. First, the laser assembly is set to a steady 20 °C temperature with all thermo-optic phase shifters turned off, and the amplifier current is set to about 65 mA. Subsequently, the phase shifter on ring resonator 1 is tuned to maximize the laser output power. After, the phase shifter on the tunable outcoupler (MZI) and the phase section are iteratively optimized to maximize the laser output power. Typically, the laser is now set at the peak gain wavelength and emits maximum output power for the set current. In the next step the laser is coarsely tuned close to, and just shorter than, the wavelength of interest by tuning the phase shifter on ring resonator 1. Adding a little bit of phase to ring resonator 1, observe that the laser mode hops beyond the wavelength of interest. Subsequently the phase on ring 2 is increased until the laser wavelength hops below the wavelength of interest. Increase the phase on ring 1 to bring the wavelength up. Repeat the heating of both ring resonators iteratively until the laser wavelength is within about 100 GHz of the wavelength of interest. Together with temperature tuning of the assembly (maximum ±3 °C) and tuning all three heaters now set the laser to the wavelength of interest as follows. First tune the phase section with ring 1 and 2 in a ratio of 5.2:1 in small steps to find the upper wavelength edge of the desired mode-hop-free tuning window. Once reached, increase the TEC temperature by 0.002-0.1 °C, this will move the entire mode-hop-free tuning window up in wavelength. Note, there will be some hysteresis when tuning the heaters and the temperature element. Stop tuning the assembly temperature until the center of the mode-hop-free tuning range is at the desired set wavelength. If any of the heaters reach an upper limit for the controller, tune them in phase down by 2π.

A recording of the laser frequency over time, with the laser set at the $^{88}Sr^{+}$ $P^{3/2} \rightarrow S^{1/2}$ transition frequency (ν = 734.99068 THz) is shown in Supplementary Fig. 2c. A recording with no manual heater control, blue line, shows a passive stability of the laser where it averages around the 22.1 MHz wide transition. An automated control loop is not yet implemented, but a manual control is explored in Supplementary Fig. 2c as well (purple line). Here, the + and - markers denote when the phase section heater was adjusted 0.15 mW in the positive and negative direction, respectively.

Short-term frequency noise

Delayed self-heterodyne measurements, following the approach described in [3], are carried out with a fiber delay length of 7.6 m (S405-XP fiber), an acousto-optic modulator (at 200 MHz, G&H FiberQ model S-M200-0.4C2A-3-F2P) and a avalanche photodiode (Thorlabs APD430A2), with signals being recorded with an oscilloscope (Agilent MSO6104a). To match the polarization of the signal from both arms of the set-up, a fiber polarization controller is used (Thorlabs FPC560 with an S405-XP fiber). A total of 10 traces with a 1-ms duration are recorded at a 4 GSa/s rate. Each trace is subsequently analyzed to obtain the frequency noise power spectral density, which starts by performing a Hilbert transform on the signal to retrieve the instantaneous phase. After unwrapping the phase the first order time derivative is calculated to obtain the instantaneous frequency, from which the carrier frequency (here, 200 MHz) is subtracted. To obtain the frequency noise PSD of a single recording with a low variance at high frequency offsets, the discrete Fourier transform of the instantaneous frequency (periodogram) is calculated with different window sizes. For the first periodogram (of 1 ms duration) the entire recording is Fourier transformed, setting the lower bound frequency offset at 1 kHz. For the subsequent periodogram, the recording is split in two equal, non-overlapping parts and the periodograms of each segment of 0.5 ms duration is calculated and both are averaged. This results in a periodogram starting at 2 kHz with a lower variance. For each following periodogram the window size is halved with respect to the previous periodogram. This process is repeated until a total of 7 periodograms are calculated, with the final

periodogram averaged over 64 segments. The 7 periodograms are finally combined. The next step is to apply a correction function to reduce the fringe effects at the Mach-Zehnder FSR frequencies of the delayed self-heterodyne set-up [4]. The analyzed frequency noise PSD of all 10 recordings are averaged. In the final step the data points at the first few offset frequencies, from 1 to 5 kHz, are removed due to high standard deviation error. The oscilloscope limits a trace duration of 1-ms at the 4 GSa/s rate, which prevents a high accuracy Fourier transform in the lower kHz frequency range.

Supplementary Note 6. Calculation of intrinsic laser linewidth

We use a theoretical approximation of the linewidth as derived in previous work [5] and adapt it to the specifics of the laser presented here. All the parameters used in the formulas below are to replicate the experimental conditions and parameters for the linewidth measurement as shown in Fig. 2f (main text) and can be found in Supplementary table 1. The adapted, Schawlow-Townes laser linewidth is described as [6] (Eq. 1)

$$\Delta\nu_{\mathrm{ST}} = \frac{1}{4\pi} \cdot \frac{v_g^2 h\nu n_{\mathrm{sp}} \gamma_m \gamma_{\mathrm{tot}} (1+\alpha_H^2)}{P_{\mathrm{out,tc}} \eta_P} \cdot \frac{\alpha_P}{F^2}. \tag{1}$$

Here, $v_g = c/n_{\mathrm{g,gain}}$ is the group velocity in the gain section with c the speed of light and $n_{\mathrm{g,gain}}$ the group index, $h\nu$ is the photon energy using, $\nu = c/\lambda_0$, and λ_0 the vacuum wavelength. In Eq. 1, n_{sp} is the population inversion factor, i.e., which specifies the ratio between the spontaneous rate of downward transitions and the stimulated rate of downward and upward transitions [7], [8]. Henry's linewidth enhancement factor is denoted by α_H, describing the strength of the so-called gain-index coupling in the gain section which is a special difference between diode lasers and simple four level lasers, e.g., solid state of gas lasers [9]. The Petermann factor is denoted by α_P [10] and accounts for the noise from amplified spontaneous emission due to the large, and asymmetric outcoupling. The linewidth reduction factor F represents a linewidth reduction from the extension of the cavity length which increases the photon lifetime. Additionally it captures the rapid change in transmission of the Vernier filter around a resonance, which favors the red detuned (falling slope) side of the Vernier resonance for obtaining the lowest linewidths. The type of lasers discussed here have various output channels, however, for this particular case the waveguide after the tunable outcoupler denoted by *output* in Fig. 1b (main text) is used to measure the laser output power. The previous description of the Schawlow-Townes linewidth [5] uses the output power measured at the back facet of the amplifier waveguide. The adapted description we use here [6], converts the on-chip power directly after the tunable coupler $P_{\mathrm{out,tc}}$ to the power at the back facet of the amplifier waveguide by multiply $P_{\mathrm{out,tc}}$ with a conversion factor η_P. Coming back to the first equation (1), the so-called distributed mirror loss coefficient γ_m in Eq. 1 is described as

$$\gamma_m = \frac{-1}{2L_g} \ln[R_b R_o]. \tag{2}$$

Here, R_b is the reflectance of the backfacet of the gain section (here amplifier) and R_o can be expressed as $R_o = 1 - T_{out}$ with T_{out} the fraction of light that is coupled out of the circuit via the output waveguide. For the particular laser described here the outcoupling, and thus R_o, can be varied using the tunable outcoupler consisting of a Mach-Zehnder interferometer (see Supplementary Fig. 1b). In Eq. 2, L_g is the geometric length of the gain section. The total distributed loss in the laser cavity is described by γ_{tot}, which becomes

$$\gamma_{\mathrm{tot}} = \frac{-1}{2L_g} \ln[R_b R_o T_g^2 T_c^2 T_F]. \tag{3}$$

Here, T_g is the transmission of the gain section reduced by internal losses in the gain section [11], described as $T_g = e^{-\gamma_g L_g}$. The squared value of T_g in Eq. 3 originates from the double-pass

through the gain section in a single cavity roundtrip. The optical loss per unit length in the gain section is denoted by γ_g. All the data regarding the amplifier in Supplementary table 1 were supplied by the manufacturer EXALOS AG. The transmission of the optical mode at the amplifier and feedback chip interface is T_c, limited by coupling losses, scattering and alignment error. This transmission factor is also squared in Eq. 3 because the light passes this coupling interface twice per roundtrip. T_F is the transmission through the feedback chip, with $T_F = T_{\text{bus}} T_{\text{P,Vernier,drop}}$. Here $T_{\text{bus}} = 10^{-\alpha_{\text{p}} L_{\text{bus}}/10}$ is the transmission through the bus waveguides in the feedback circuit, where α_{p} (in dB per unit length) is the optical loss in the Al_2O_3 waveguides and L_{bus} is the geometric length of the bus waveguides in a single roundtrip.

$T_{\text{P,Vernier,drop}}$ is the frequency dependent, power transmission through the Vernier filter at the drop port back to the amplifier given by:

$$T_{\text{P,Vernier,drop}} = |T_{\text{E,R}_1\text{,drop}}|^2 \cdot |T_{\text{E,R}_2\text{,drop}}|^2 \tag{4}$$

Here $T_{\text{E,R}_1\text{,drop}}$ and $T_{\text{E,R}_2\text{,drop}}$ are the field transmission coefficients of respectively the sequentially coupled micro-ring resonators R_1 and R_2 at the respective drop ports. The field transmission to the drop port of each ring can be written as [12]:

$$T_{E,R_i,drop} = \frac{-\kappa^2 \sqrt{\chi_r} e^{i\phi_r/2}}{1 - t^2 \chi_r e^{i\phi_r}} \tag{5}$$

The field cross and bar coefficients of the directional couplers between the bus and ring waveguides are represented by κ and t, respectively. We assume loss-less couplers, expressed as $\kappa^2 + \text{t}^2 = 1$ and we assume symmetric coupling at the through and drop port. The roundtrip phase in the ring resonator is described as $\phi_r = 2\pi^2/\lambda R_i n_{\text{eff}}$, here R_i is the ring resonator radius and n_{eff} is the effective mode index. The dispersion of the effective mode index, from which also the group index is calculated, can be described by a linear approximation $n_i = n_0(1 - n_a\lambda)$ with the variables n_0 and n_a. The loss factor per roundtrip is described by $\chi_r = 10^{-\alpha_{\text{prop}} 2\pi R_i n_{\text{eff}}/20}$.
$P_{\text{out,tc}}$, in Eq. 1, is the on-chip, optical output power of the laser right after the tunable coupler (see Fig. 1b, main text). Here, to obtain $P_{\text{out,tc}}$, we take the on-chip power at the end facet P_{out} and backpropagate it using the propagation loss with $P_{\text{out,tc}} = P_{\text{out}} \cdot 10^{\alpha_p L_{\text{out}}}$. Here, L_{out} is the geometric length of the bus waveguide from the tunable coupler to the end facet. As touched upon before, in order to let the linewidth predicted by Eq. 1 refer to the laser power emitted through the Al_2O_3 output waveguide instead of the back facet of the semiconductor waveguide as in [5], the denominator also contains a conversion factor, η_p given by [6]:

$$\eta_P = 1 + \frac{1 - R_b}{1 - R_o} \sqrt{\frac{R_o}{R_b}} \frac{\sqrt{T_b^{\rightarrow} T_b^{\leftarrow}}}{\sqrt{T_b^{\rightarrow}}}. \tag{6}$$

Here, $T_b^{\rightarrow}$ is the counter-clockwise cavity transmission of the Al_2O_3 waveguides starting at the front facet with the amplifier, to and looping through the Vernier filter until the left directional coupler in the tunable outcoupler. Which can be described by $T_b^{\rightarrow} = T_{\text{P,Vernier,drop}} \cdot 10^{-\alpha_p L_b^{\rightarrow}}$ with $L_b^{\rightarrow}$ the geometric length of the bus waveguides in the path described. In Eq. 6 $T_b^{\leftarrow}$ is the clockwise cavity transmission of the Al_2O_3 waveguides starting at left directional coupler in the tunable outcoupler, to the front facet with the amplifier, described by $T_b^{\leftarrow} = 10^{-\alpha_p L_b^{\leftarrow}}$ with $L_b^{\leftarrow}$ the geometric length of the bus waveguides in the path described. We recall that the Petermann factor α_P [10] in Eq. 1 describes the extra phase noise due to amplified spontaneous emission coupling into the laser mode from the large and asymmetric outcoupling in the cavity. The factor α_P is calculated using [13]:

$$\alpha_P = \left| \frac{(r_1 + r_2)(1 - r_1 r_2)}{2 r_1 r_2 \ln(r_1 r_2)} \right|^2, \tag{7}$$

With $r_1 = \sqrt{R_b}$ and $r_2 = \sqrt{R_o T_F T_c^2}$. Here, α_P is wavelength dependent through the frequency selective transmission T_F of the Vernier filter.
We recall factor F in Eq. 1 as a linewidth reduction factor, where $F = 1 + A + B$. With, A as

the linewidth narrowing through extension of the cavity length and B through the dispersion of the frequency dependent (Vernier filter) feedback circuit. Here, A is given by [5]:

$$A = \frac{1}{\tau_g}\left(\frac{d\phi_{\text{feedback}}}{d\omega}\right). \tag{8}$$

The roundtrip time in the gain section is $\tau_g = 2n_{\text{g,gain}}L_g/c$, with $n_{\text{g,gain}}$ the group index of the gain section and $\phi_{\text{feedback}} = \phi_{\text{bus}} + \phi_V$ is the accumulated phase of the light (as a function of frequency f or angular frequency $\omega = 2\pi f$) from a roundtrip in the feedback chip. $\phi_{\text{bus}} = 2\pi n_{\text{eff}}L_{\text{bus}}/\lambda$ denotes the accumulated phase of the light in the bus waveguides of the circuit. The phase accumulation caused by the Vernier filter (ϕ_V) is the sum of the phase accumulated in the individual ring resonators. The phase accumulation due to a single ring can be found by unwrapping the polar angle of the complex field transmission t_{R_i} at the ring resonator drop port. The term B in the linewidth reduction factor F is given by [5]:

$$B = \frac{\alpha_H}{\tau_g}\left(\frac{d\ln|t_F|}{d\omega}\right), \tag{9}$$

Here t_F is the field transmission through the feedback chip, $t_F = \sqrt{T_F}$. The term B can contribute to linewidth narrowing, especially for micro-ring resonator based extended cavity lasers, due to the strong frequency dependent transmission in the Vernier filter. This helps stabilize the laser frequency, often referred to as *detuned loading* [14], and will be explained in more detail here. The feedback chip transmission t_F varies strongly around a Vernier filter resonance, as can be seen in Fig. 2a (main text). At the red frequency detuned side of the Vernier resonance, the slope of the filter transmission, and thus t_F, as function of optical frequency is positive. The result is that, for example, any small frequency detuning in the positive direction will increase the feedback chip transmission t_F and reflectivity back to the semiconductor amplifier. Here the increase in intensity will reduce the carrier density, i.e., gain. Due to the aforementioned gain-index coupling in the semiconductor waveguide denoted by Henry's factor, which is taken as 5 here (Supplementary table 1), the index will increase strongly. This shifts the cavity resonance to a lower frequency, effectively dampening the initial shift to a higher frequency and narrowing the linewidth. At the blue frequency detuned side of the Vernier resonance the $\delta t_F/\delta f$ is negative and the same effect works to destabilize and actively broaden the linewidth.

Using the expressions above in eq. 1 and inserting the parameters shown in Supplementary table 1 (including the errors for four parameters), the intrinsic laser linewidth $\Delta\nu_{\text{ST}}$ is calculated to be 92 kHz at minimum for the case that the laser cavity mode is perfectly aligned with the center of the Vernier resonance. However, as discussed, the linewidth narrowing factor F (with A and B) depends strongly on where wavelength of the laser is tuned with respect to the Vernier filter resonant wavelength, as shown in Supplementary Fig. 3a. Experimentally the laser can be frequency detuned via the heater at the phase section (see Fig. 1b main text). During the experiment this heater is optimized until the minimum intrinsic linewidth is found, and at this setting also the output power is measured. The exact frequency detuning is difficult to determine since the phase section causes a phase shift, originating from thermal cross-talk, to the light in the Vernier filter. By plotting the intrinsic linewidth as a function of detuning the laser with respect to the Vernier resonance we find a minimum intrinsic linewidth of $\Delta\nu_{ST,min} = 63$ kHz, shown in Supplementary Fig. 3b. Although, the laser output power varies slightly with different detuning this is not taken into account for calculating Supplementary Fig. 3b. The experimental result is not far from the fundamental limit of the laser linewidth expressed by eq. 1.

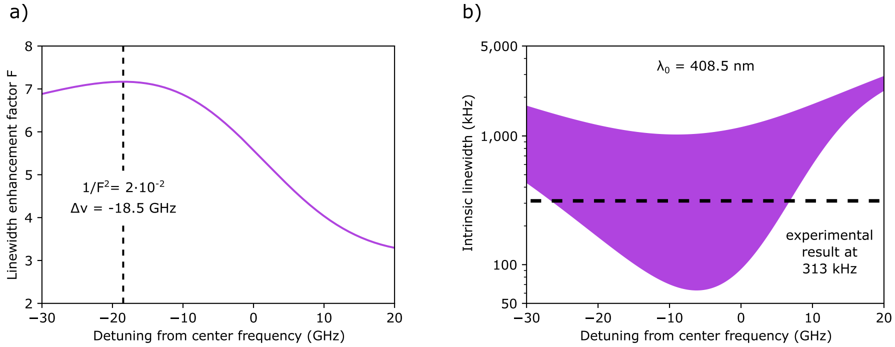

Supplementary Figure 3: **a)** Linewidth narrowing factor F as a function of detuning the laser from the main Vernier resonant frequency. At -18.5 GHz the linewidth narrowing factor is strongest, the factor $1/\mathrm{F}^2$ in eq. 1 is here $2 \cdot 10^{-2}$. For this calculation the error in the last four parameters of Supplementary table 1 are not taken into account. **b)** The violet shaded area indicates the calculated intrinsic linewidth as a function of laser detuning, taking the error of the last four parameters in Supplementary table 1 in consideration. With zero detuning the intrinsic linewidth is 92 kHz at minimum while the ultimate limit is achieved at 63 kHz by red detuning the laser frequency. The horizontal dashed line indicates the measured linewidth for comparison, see Fig. 2f (main text).

Parameter	Value	Min. value	Max. value
λ_0 (nm)	408.5	-	-
n_{sp}	1	-	-
α_H	5	-	-
$L_g(\mu m)$	600	-	-
$\gamma_g(m^{-1})$	500	-	-
R_b	0.95	-	-
T_{out}	0.37	-	-
$n_{\mathrm{g,gain}}$	3.59	-	-
$n_{\mathrm{0,eff}}$	1.804	-	-
$n_{\mathrm{a,eff}}(\mu m^{-1})$	0.367	-	-
$n_{\mathrm{0,g}}$	2.541	-	-
$n_{\mathrm{a,g}}(\mu m^{-1})$	0.725	-	-
Bus total cavity waveguide length L_{bus} (mm)	8.66	-	-
Bus output waveguide length L_{out} (mm)	7.40	-	-
Bus counter-clockwise waveguide length $L_b^{\rightarrow}$ (mm)	6.58	-	-
Bus clockwise waveguide length $L_b^{\leftarrow}$ (mm)	2.08	-	-
Radius MRR 1 R_1 (μm)	150	-	-
Radius MRR 2 R_1 (μm)	153	-	-
α_{p} (dB/cm)	2.86	2.56	3.16
κ^2	0.58	0.44	0.71
T_c	0.31	0.19	0.51
P_{out} (mW) (in on-chip output waveguide at facet)	1.42	0.88	2.30

Supplementary Table 1: Model parameters to obtain the intrinsic linewidth.

Supplementary Note 7. Calculation of the thermorefractive noise

Previous work has shown that thermorefractive noise can be a dominant thermal noise source in the frequency stability micro-ring resonators [15], [16]. In this section we will estimate the thermorefractive noise of the extended cavity caused by the feedback chip to estimate if such noise at high offset frequencies is limiting the laser frequency noise or if the laser linewidth is given by the Schawlow-Townes limit, as discussed in the previous section.
In the first step we calculate thermorefractive noise of the first micro-ring resonator in the vernier filter. To this end a finite element method (FEM) simulation is performed in COMSOL to obtain the thermorefractive noise $S_{\delta f_{R1}}$ of ring resonator 1. The method is used is described in the main and supplementary text by Huang et al.[15]. The calculation is performed at a resonance frequency of $f_{R1} = c/\lambda_0$ with $\lambda_0 = 408.5$ nm, similar to the laser setting used in the linewidth measurement shown in Fig. 2f (main text). The waveguide geometry used is the same as described in the main text, with a bottom and top cladding of 8 μm SiO_2, Si substrate and air background. The other parameters used for this FEM simulation are shown in Supplementary table 2. The calculated thermorefractive noise $S_{\delta f_{R1}}$ for ring resonator 1 is displayed in Supplementary Fig. 4.
To find the thermorefractive noise of the entire laser cavity, we first recognize the separate components that constitute the laser cavity. First, the laser cavity consists of three sub-resonators, which are the two micro-ring resonators and the sub-resonator formed by the bus waveguides. The latter also contains the GaN amplifier waveguide. However, currently there are no detailed data on the cross-sectional geometry, material indices and thermal properties for this commercial amplifier available that would allow us to calculate its thermorefractive noise contribution. However, for our laser we estimate that the contribution of the GaN amplifier to the thermorefractive noise is minor (∼1 dB), due to it small contribution to the laser cavity mode volume (5%), even when the thermo-optic coefficient ($2.3 \cdot 10^{-4}$ [17]) higher, compared to Al_2O_3. The thermorefractive noise for the other two cavity contributions, i.e., ring resonator 2 and the bus waveguides, can be obtained by scaling $S_{\delta f_{R1}}$ with the ratio of the cavity mode volume to the mode volume of ring resonator 1. Since the waveguide cross-section remains the same throughout all structures we can assume to only scale with the length, such that $S_{\delta f_{R2}} = S_{\delta f_{R1}}/(R_1/R_2)$ and $S_{\delta f_{bus}} = S_{\delta f_{R1}}/(2\pi R_1/L_{bus})$, also shown in 4. After having introduced the various cavity components, we need to realize that the laser cavity cannot be treated as a single volume where thermorefractive noise has the same effect on the laser frequency noise, wherever this noise arises along the laser cavity. The reason is, as described in literature, that the laser wavelength variation is different for phase variations occurring in the bus waveguides compared to the ring resonators [18]. This is directly related to the different FSR of the ring resonator based Vernier filter and the bus waveguide cavity. This difference needs to be taken into account as weight factors when the full laser cavity thermorefractive noise is calculated. To obtain a relation between the sensitivity of the laser frequency variation with regard to phase variations in the different contributing cavity elements, we first extend our current model for the transmission and modes of the ring resonators and Vernier filter, by including bus waveguides as well. First the roundtrip phase of the laser cavity is calculated, defined as

$$\phi_{RT} = \phi_g + \phi_{bus} + \phi_V \tag{10}$$

Here, the phase contribution from the gain section $\phi_g = 2\pi n_{\text{eff,g}} L_g/\lambda$ and $n_{\text{eff,g}}$ can be calculated using $n_{0,\text{eff,g}}$ $n_{\text{a,eff,g}}$ in tabel 2 and the same linear relation as discussed in the previous section. The other phase contributions have been discussed in the previous section. The reflection function describing the modes in the laser cavity can then be obtained as (adapted from [19])

$$R_c = 1 - \frac{1}{1 + (2F_c/\pi)^2 \sin^2(\phi_{RT})} \tag{11}$$

Here F_c is the Finesse of a cavity given by

$$F_c = \frac{\pi\sqrt{r_c}}{1 - r_c} \tag{12}$$

In this expression, the recirculation coefficient of the field amplitude in the laser, r_c, is given as

$$r_c = R_b R_0 T_F T_g^2 T_c^2 \tag{13}$$

The other variables have been introduced above. The sensitivity of the laser wavelength can be numerically calculated by measuring the laser frequency shift δf_{laser}, or frequency at the peak value of R_c, as a function of phase shift $\delta\phi$ for each contributing structure. Before this resonance sensitivity is evaluated we add a phase constant for each structure that centers all cavity resonances to the wavelength of interest, λ_0. This also makes sure that the phase in the bus waveguides is optimally aligned with the resonances of both rings, similar to what we do experimentally by adjusting the on-chip heaters. The evaluated cavity sensitivities (f'_{R1}, f'_{R2}, f'_{bus}) can be found in Supplementary table 2. Using these, the total thermorefractive frequency noise of the laser is found as:

$$S_{\delta f_{\text{laser}}} = {f'_{R1}}^2 S_{\delta\theta_{R1}} + {f'_{R2}}^2 S_{\delta\theta_{R2}} + {f'_{bus}}^2 S_{\delta\theta_{bus}} \tag{14}$$

Here $S_{\delta\theta_i}$ denotes the phase noise of each contributing structure, since the sensitivity factors are defined as a laser frequency shift δf_{laser} versus phase shift $\delta\phi$. As the pre-factors f'_i relate to the amplitude of the noise, they are squared in the summation of the power spectral densities. We express the phase noise of a cavity as

$$S_{\delta\theta i} = \frac{4 S_{\delta f_i}}{\kappa_{c,i}^2} \tag{15}$$

The full width, half maximum linewidth of each cavity is $\kappa_{c,i}$ can be found numerically, with all values shown in Supplementary table 2. The calculated, full laser cavity thermorefractive noise $S_{\delta f_{\text{laser}}}$ is shown in Supplementary Fig. 4. As expected, due to the larger mode volume of the combined resonator components, the laser cavity thermorefractive noise is lower than the noise of each individual component.

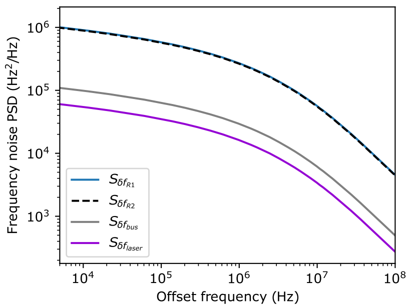

Supplementary Figure 4: Calculated thermorefractive noise of ring resonator 1 ($S_{\delta f_{R1}}$), ring resonator 2 ($S_{\delta f_{R2}}$), the resonator contribution from the bus waveguides ($S_{\delta f_{bus}}$), and of the full laser cavity ($S_{\delta f_{laser}}$).

Parameter	Value
λ_0 (nm)	408.5
T (K)	293
κ_T (W/(m K))	30
ρ (kg/m^3)	3.99 $\cdot 10^3$
C (J/(kg K))	779
dn/dT_{clad} (K^{-1})	0.95 $\cdot 10^{-5}$
dn/dT_{core} (K^{-1})	4.92 $\cdot 10^{-5}$
$n_{Al_2O_3}$ (at λ_0)	1.756
n_{SiO_2} (at λ_0)	1.479
$n_{0,\mathrm{eff,g}}$	3.644
$n_{\mathrm{a,eff,g}}$	0.752
f'_{R1} (GHz/rad)	-3.19
f'_{R2} (GHz/rad)	-3.24
f'_{bus} (GHz/rad)	-2.05
$\kappa_{c,R1}$ (GHz)	44.7
$\kappa_{c,R2}$ (GHz)	43.9
$\kappa_{c,bus}$ (GHz)	10.0

Supplementary Table 2: Parameters for the thermorefractive noise calculations.

References

[1] C. I. van Emmerik, W. A. P. M. Hendriks, M. M. Stok, et al. " Relative oxidation state of the target as guideline for depositing optical quality RF reactive magnetron sputtered Al_2O_3 layers ". *Optical Materials Express* 10.6 (2020). DOI: 10.1364/ome.393058.

[2] S. Jétte-Charbonneau, N. Lahoud, R. Charbonneau, et al. "End-facet polishing of surface plasmon waveguides in lithium niobate". *IEEE Transactions on Advanced Packaging* 31.3 (2008). DOI: 10.1109/TADVP.2008.924236.

[3] L. E. Richter, H. I. Mandelberg, M. S. Kruger, et al. "Linewidth Determination From Self-Heterodyne Measurements With Subcoherence Delay Times." *IEEE Journal of Quantum Electronics* 22.11 (1986). DOI: 10.1109/jqe.1986.1072909.

[4] Z. Yuan, H. Wang, P. Liu, et al. "Correlated self-heterodyne method for ultra-low-noise laser linewidth measurements". *Optics Express* 30.14 (2022). DOI: 10.1364/oe.458109.

[5] K. J. Boller, A. van Rees, Y. Fan, et al. "Hybrid integrated semiconductor lasers with silicon nitride feedback circuits". *Photonics* 7.1 (2020). DOI: 10.3390/photonics7010004.

[6] A. van Rees. "Widely-tunable and ultra-stable hybrid-integrated diode lasers". PhD thesis. Enschede, The Netherlands: University of Twente, 2024. DOI: 10.3990/1.9789036558952.

[7] G. Lasher and F. Stern. "Spontaneous and Stimulated Recombination Radiation in Semiconductors". *Physical Review* 133.2A (1964). DOI: 10.1103/PhysRev.133.A553.

[8] H. Wenzel, M. Kantner, M. Radziunas, et al. "Semiconductor Laser Linewidth Theory Revisited". *Applied Sciences* 11.13 (2021). DOI: 10.3390/app11136004.

[9] C. Henry. "Theory of the linewidth of semiconductor lasers". *IEEE Journal of Quantum Electronics* 18.2 (1982). DOI: 10.1109/JQE.1982.1071522.

[10] K. Ujihara. "Phase noise in a laser with output coupling". *IEEE Journal of Quantum Electronics* 20.7 (1984). DOI: 10.1109/JQE.1984.1072472.

[11] A. Castiglia, M. Malinverni, C. Mounir, et al. "Recent progress on GaN-based superluminescent light-emitting diodes in the visible range". *SPIE OPTO* (2018). DOI: 10.1117/12.2287763.

[12] E. J. Klein. *Densely integrated microroring-resonator based components for fiber-to-the-home applications.* PhD thesis, University of Twente, 2007. ISBN: 9789036524957.

[13] Y. Fan, R. E. M. Lammerink, J. Mak, et al. "Spectral linewidth analysis of semiconductor hybrid lasers with feedback from an external waveguide resonator circuit". *Optics Express* 25.26 (2017), p. 32767. ISSN: 1094-4087. DOI: 10.1364/oe.25.032767.

[14] M. A. Tran, D. Huang, and J. E. Bowers. "Tutorial on narrow linewidth tunable semiconductor lasers using Si/III-V heterogeneous integration". *APL Photonics* 4.11 (Nov. 2019). DOI: 10.1063/1.5124254.

[15] G. Huang, E. Lucas, J. Liu, et al. "Thermorefractive noise in silicon-nitride microresonators". *Physical Review A* 99.6 (2019). DOI: 10.1103/PhysRevA.99.061801.

[16] N. Kondratiev and M. Gorodetsky. "Thermorefractive noise in whispering gallery mode microresonators: Analytical results and numerical simulation". *Physics Letters A* 382.33 (2018). DOI: 10.1016/j.physleta.2017.04.043.

[17] N. Watanabe, T. Kimoto, and J. Suda. "Thermo-optic coefficients of SiC, GaN, and AlN up to 512°C from infrared to ultraviolet region for tunable filter applications". *Micromachining and Microfabrication Process Technology XVI.* Vol. 7926. 2011. DOI: 10.1117/12.874531.

[18] A. van Rees, Y. Fan, D. Geskus, et al. "Ring resonator enhanced mode-hop-free wavelength tuning of an integrated extended-cavity laser". *Optics Express* 28.4 (2020). DOI: 10.1364/OE.386356.

[19] B. Saleh. *Fundamentals of Photonics.* 2nd ed. Wiley. ISBN: 978-0-471-35832-9.
